# Impact of microscopic In fluctuations on the optical properties of In_*x*_Ga_1-*x*_N blue light-emitting diodes assessed by low-energy X-ray fluorescence mapping using synchrotron radiation

**DOI:** 10.1038/s41598-019-39086-5

**Published:** 2019-03-06

**Authors:** Atsushi Sakaki, Mitsuru Funato, Munehiko Miyano, Toshiyuki Okazaki, Yoichi Kawakami

**Affiliations:** 1 Nichia Corporation, Anan, Tokushima 774-8601 Japan; 2 Department of Electronic Science and Engineering, Kyoto University, Kyoto, 615-8510 Japan

## Abstract

Among the III-nitride semiconductors, In_*x*_Ga_1-*x*_N is a key material for visible optical devices such as light-emitting diodes (LEDs), laser diodes, and solar cells. Light emission is achieved via electron-hole recombination within the In_*x*_Ga_1-*x*_N layer. When In_*x*_Ga_1-*x*_N-based blue LEDs were first commercialized, the high probability of electron-hole radiative recombination despite the presence of numerous threading dislocations was a mystery. Extensive studies have proposed that carrier localization in nanoscopic potential fluctuations due, for example, to the immiscibility between InN and GaN or random alloy fluctuations is a key mechanism for the high emission efficiency. In actual LED devices, not only nanoscopic potential fluctuations but also microscopic ones exist within the In_*x*_Ga_1-*x*_N quantum well light-emitting layers. Herein we map the synchrotron radiation microbeam X-ray fluorescence of In_*x*_Ga_1-*x*_N blue LEDs at a sub-micron level. To acquire weak signals of In, Ar, which is in the air and has a fluorescent X-ray energy similar to that of In, is evacuated from the sample chamber by He purge. As a result, we successfully visualize the spatial In distribution of In_*x*_Ga_1-*x*_N layer nondestructively and present good agreement with optical properties. Additionally, we demonstrate that unlike nanoscopic fluctuations, microscopic In compositional fluctuations do not necessarily have positive effects on device performance. Appropriately controlling both nanoscopic and microscopic fluctuations at the same time is necessary to achieve supreme device performance.

## Introduction

In_*x*_Ga_1-*x*_N is an alloy composed of InN and GaN, and a key material for visible light-emitting or detecting devices because the bandgap can be adjusted between 3.4 eV (365 nm wavelength) and 0.6 eV (2.1 μm) by changing the In composition *x*^1^. Previously, realizing blue light-emitting diodes (LEDs) was challenging due to the difficulty of preparing the In_*x*_Ga_1-*x*_N layer itself^2,3^. Although blue LEDs have been commercialized for over a quarter of a century, problems related to In_*x*_Ga_1-*x*_N emitters such as temperature characteristics (efficiency reduction when the junction temperature rises) and droop phenomenon (efficiency reduction at high current driving) remain.

One important factor that determines the optical properties of In_*x*_Ga_1-*x*_N quantum well (QW) light emitters is the immiscibility between InN and GaN caused by the large lattice mismatch^4^. The immiscibility may induce In compositional fluctuations and consequently potential fluctuations, affecting the carrier recombination dynamics within In_*x*_Ga_1-*x*_N QWs. Optically, such potential fluctuations have been assessed using time-resolved photoluminescence (TRPL) spectroscopy^5^, scanning near field optical microscopy^6,7^, and cathodoluminescence (CL) spectroscopy^8^. Structurally, transmission electron microscopy (TEM)^9^ and atom probe tomography (APT) are conventionally used for high-resolution nanoscopic analyses. It is noteworthy that In_*x*_Ga_1-*x*_N QWs are embedded in GaN matrices, and cross sections are typically observed. Thus, the measurements are destructive. It is also difficult to evaluate the in-plane two-dimensional distribution of In over a wide area. Furthermore, electron beam irradiation during TEM observations may cause the In atoms in the In_*x*_Ga_1-*x*_N layer to move^10,11^.

An X-ray microbeam based on synchrotron radiation (SR) is a nondestructive option for structural analyses, though its spatial resolution is worse than TEM. Recently, we demonstrated a clear correlation between the In_*x*_Ga_1-*x*_N spatial distribution and the luminescence inhomogeneity using X-ray microbeam diffraction and CL^12^. A sub-micron spatial resolution is achieved in the direction perpendicular to the incident plane, but it is enlarged within the incident plane to ~10 μm due to the inclined incident of the X-ray beam at the Bragg angle. To achieve a higher resolution, SR microbeam X-ray fluorescence (XRF) imaging seems promising because the X-ray can be incident at the surface normal. To date, several papers have reported XRF of In in In_*x*_Ga_1-*x*_N quantum structures using SR facilities^13–16^. However, high-resolution structural and optical assessments at the same position have yet to be achieved in a realistic device structure.

In this study, we demonstrate a clear correlation between the local In distributions and the optical properties in blue-emitting In_*x*_Ga_1-*x*_N LEDs using the XRF technique with a sub-micron SR X-ray beam and a PL mapping technique. In addition, we show that the SR XRF technique is applicable not only to planar LED structures but also to three-dimensional (3D) structures suitable for polychromatic emission^17–22^. One may feel that the combination of scanning electron microscopy (SEM) and energy dispersive X-ray spectrometry (EDX) is handier for analyses of the local In distributions. However, surface coating with a conductive film is often necessary. In addition, due to the spread of irradiated electrons within the material, the spatial resolution of SEM-EDX is limited to a few hundreds of nm, which is one order worse than that of state-of-the-art SR-XRF^13–15^. Therefore, SR XRF enables more detailed analyses.

Here we briefly review previous studies on SR XRF applied to In_*x*_Ga_1-*x*_N. In-rich In_*x*_Ga_1-*x*_N nanowires have been investigated using the ID22NI beam line of the European Synchrotron Radiation Facility (ESRF)^13–15^. One characteristic of the In-rich In_*x*_Ga_1-*x*_N nanowires is significant spatial In compositional distributions ranging from 0.30 to 0.95 induced by the nano-structure effect^13^. The X-ray beam was focused by a pair of Kirkpatrick-Baez (KB) mirrors to a size of ~50 × 50 nm^2^ to detect the In fluorescence X-ray^13–15^. The monitored X-ray lines were the In *K*α line with an irradiated X-ray energy of 29.6 keV^13^, the In *L*α line with an irradiated X-ray energy of 17.0 keV^14,15^. The nanoprobe system realized a very good spatial resolution, but the correlation between the optical and structural properties was not evaluated. Miyajima *et al.* characterized a green-emitting In_0.2_Ga_0.8_N QW using the BL37XU beam line of the Super Photon ring-8 GeV (SPring-8)^16^. The X-ray beam was focused similarly by a pair of KB mirrors to a size of ~1.3 × 3.8 μm^2^. The In *K*α line was monitored with an irradiated X-ray energy of 37.0 keV. To enhance the In spatial fluctuations, post-growth annealing was employed. As a result of partial In evaporation, two distinct regions, In-rich and In-poor regions are intentionally created in a μm scale. Consequently, the samples examined to date were In-rich In_*x*_Ga_1-*x*_N nanowires and annealed In_0.2_Ga_0.8_N QWs, both of which exhibit significant spatial distributions of In atoms. On the other hand, the In fluctuations should be much smaller in practically important devices operating in the blue-to-green spectral range, and SR XRF evaluations of such devices have yet to be reported. In this study, we evaluate blue LED epitaxial layers using SR XRF.

## Synchrotron Radiation for XRF

To analyze small variations in the XRF signals emitted from a small volume defined by the QW width and X-ray microbeam size, a SR facility should have a high brightness and a low divergence light source. This study employed beamline BL16XU at SPring-8 with a microbeam fabricating system.

**Figure 1:**
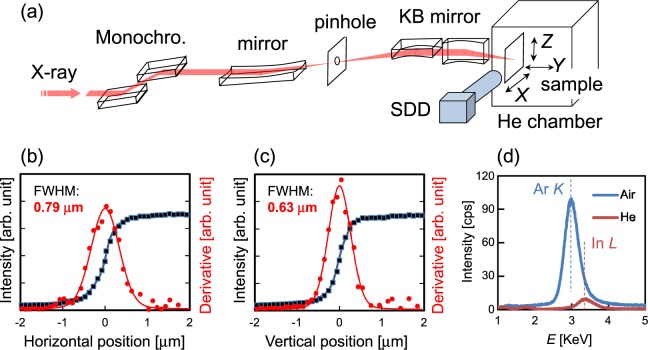
(**a**) Schematic diagram of the measurement system, (**b**) beam profile evaluated by the knife-edge method in the horizontal direction, (**c**) that in the vertical direction, and (**d**) XRF profile in a low-energy region, which shows the effect of the He atmosphere. Dashed lines represent the peak energies of Ar *K* (~2.96 keV) and In *L* (~3.29 keV).

Figure 1(a) shows a schematic diagram of the measurement system. The X-ray is monochromated to an energy as low as 9 keV by a double-crystal monochromator. Although a lower X-ray energy may lead to experimental difficulties (as discussed below), it may also provide benefits such as higher S/N ratios due to suppressed Compton scattering (as well as detectability of lightweight elements such as Al or Si in future studies). Another benefit is the selectivity of the excitation. For elements such Al, Ga, and In, which are major constituents of group III-nitride LEDs, a low-energy (9 keV) X-ray beam excites the In *L*, Al *K*, and Ga *L* lines, but not the In *K* and Ga *K* lines because the absorption edge energies of the In *K* and Ga *K* lines are higher than 9 keV. Besides, the 25-μm-thick Be window of the detector hampers detection of the Al *K* and Ga *L* lines. Because Ga is the major component element of the present LED samples, excluding Ga contributions from XRF signals remarkably facilitates the analyses of XRF maps for In, the minor component element.

The monochromated X-ray beam was focused by a front mirror onto a pinhole, which acts as a virtual light source. After passing through the pinhole, the diverging light was refocused in both the vertical and horizontal directions by a pair of KB mirrors. The focusing KB mirrors used in this study are Rh coated, and the beam incident angle to the mirror is 5 mrad. Under these conditions, the reflectivity of the mirrors is approximately 90% at an X-ray energy of 9 keV and nearly zero above 20 keV. Thus, the reflectivity is suitable for the present X-ray conditions.

The focused beam sizes evaluated by the knife-edge method are ~0.8 μm in the horizontal *X* direction and ~0.6 μm in the vertical *Z* direction (Fig. 1(b,c)). The X-ray beam focused by the KB mirrors has a higher intensity than those focused by a Fresnel zone plate (FZP) without chromatic aberrations. These features are advantageous for XRF measurements. The focused beam was irradiated on the sample with a normal incidence. Hence, the beam size determines the spatial resolution. The sample was scanned two-dimensionally in the *X*-*Z* plane. The generated XRF signal from the sample was detected by a silicon drift detector (SDD) with a detection area of 80 mm^2^ (ϕ10 mm). All measurements were performed at room temperature. The energy resolution of this measurement system cannot spectrally resolve the α and β lines of In.

It should be noted that Ar exists in air (at a content less than 1%) and the energy of the Ar *K*α line (~2.96 keV) is close to that of the In *L*α line (~3.29 keV). Therefore, when In_*x*_Ga_1-*x*_N is measured in air, the signal of In *L* XRF may be unrecognizable due to the overlap with the Ar *K* peak. To avoid such a difficulty, the sample and detector tip were set in a chamber, which was purged with He. Figure 1(d) shows the effect of the He atmosphere on the detection of low-energy XRF signals. The He atmosphere excludes the Ar peak almost completely, enabling the In *L* signal to be observed clearly.

## XRF Map of Microscopic Indium Distribution in InGaN-based Blue LEDs

**Figure 2:**
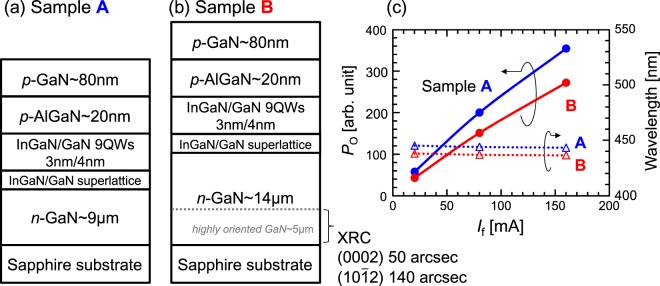
Schematic structural diagrams of Samples (**a**) A and (**b**) B. (**c**) LED characteristics of both samples as functions of the forward current *I*_f_.

This measurement system was applied to two planar LED samples A and B grown on sapphire (0001) substrates by metalorganic vapor phase epitaxy (MOVPE). Figure 2(a,b) show schematic structural diagrams of these samples, including the designed film thickness of each layer. Samples A and B were grown in succession under the same conditions in the same reactor. They consist of *n*-type GaN, In_*x*_Ga_1-*x*_N/GaN superlattices (SL), nine pairs of In_*x*_Ga_1-*x*_N/GaN multiple QWs, *p*-type AlGaN, and *p*-type GaN. (Because the amount of In in SL is less than 10% of that in the QWs, the influence of SL on the XRF signal is ignored in the following discussion).

Sample A was grown directly on a sapphire substrate whereas sample B was grown on a highly oriented, 5-μm-thick GaN template prepared in advance. As a result, the *n*-GaN thickness in Sample B (14 μm) is 5 μm thicker than that in Sample A (9 μm). The full widths at half maximums (FWHMs) of the X-ray rocking curve (XRC) of the highly-oriented GaN template are 50 arcsec for the (0002) reflection and 140 arcsec for the (10$\bar{1}$2) reflection. The XRC FWHMs of Sample A are 237 arcsec for the (0002) reflection and 241 arcsec for the (10$\bar{1}$2) reflection, whereas those of Sample B are 79 arcsec and 112 arcsec. Thanks to the highly oriented GaN template, Sample B is also highly oriented. The In compositions in the In_*x*_Ga_1-*x*_N QWs determined by the X-ray diffraction ω/2θ measurements are ~13% for Sample A and ~11% for Sample B.

Figure 2(c) shows the LED characteristics of Samples A and B as functions of the forward current *I*_f_. The output power *P*_o_ and the emission wavelength of Sample A are higher and longer, respectively, than those of Sample B. The difference of the emission wavelength is attributed to the difference in the In composition. The difference in the output power is likely due to the quality difference of the In_*x*_Ga_1-*x*_N QWs. To confirm this, low-energy XRF imaging with a SR microbeam was acquired for the buried In_*x*_Ga_1-*x*_N/GaN QWs.

**Figure 3:**
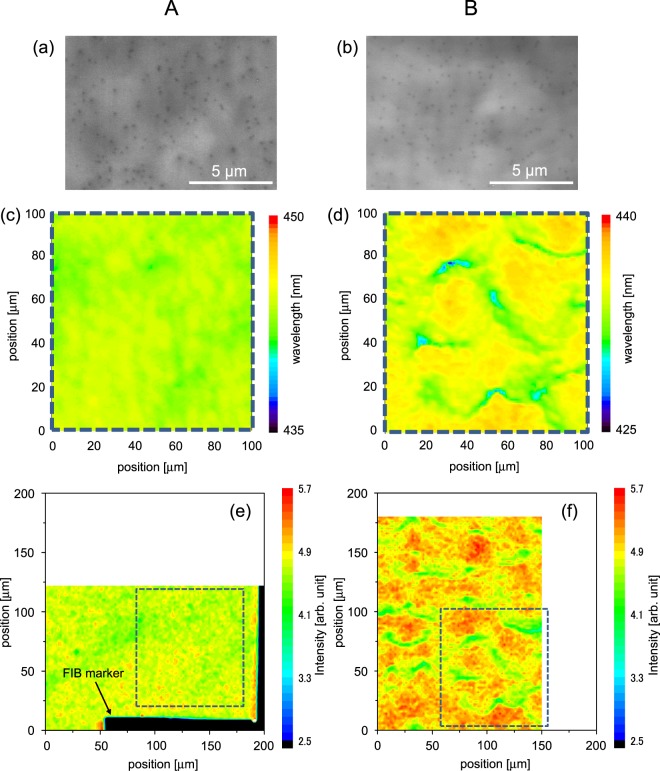
Panchromatic SEM-CL images for Samples (**a**) A and (**b**) B. (**c**,**d**) Peak wavelength distribution maps by PL and (**e**,**f**) XRF intensity maps obtained at the precisely same position with PL for Sample A [(**c**,**e**)] and Sample B [(**d**,**f**)]. Dashed squares in (**e**,**f**) correspond to the whole area in (**c**,**d**), respectively. PL wavelength range displayed on the map is set to 15 nm. Scale bars of the In XRF intensity maps are common for Samples A and B.

Figure 3(a,b) show the surface SEM–cathodoluminescence (CL) images for Samples A and B, respectively. The images are panchromatic CL images acquired at an acceleration voltage of 5 kV. Despite the large difference in the degree of the crystal tilt revealed by XRC, Samples A and B have similar dark spot densities of 1.6 × 10^8^ cm^−2^ in CL. This is probably because the samples have similar edge dislocation densities. In fact, those estimated from XRC are 3.2 × 10^8^ cm^−2^ for Sample A and 1.4 × 10^8^ cm^−2^ for Sample B.

Figure 3(c,d) show the peak wavelength distribution maps revealed by microscopic PL. The spatial resolution is ~0.5 μm, and the wavelength and output power of the excitation laser are 405 nm and 2 mW, respectively. The samples were scanned in the in-plane direction with 1-μm steps. The PL emission wavelength range displayed on the map is set to 15 nm for both samples.

Figure 3(e,f) show the XRF maps of the In *L* signals. The experimental procedure was as follows. The focused X-ray beam was normal to the incident of the sample. The sample was scanned in 2-μm steps. The acquisition time to detect the XRF signal was 5 seconds per point. The L-shaped mark in Fig. 3(e) is fabricated on the sample surface by focused ion beam (FIB), making it possible to measure PL and XRF maps at precisely the same position. The scale bar of the XRF intensity was the same between Samples A and B. Comparing Fig. 3(c,e) (Sample A), and Fig. 3(d,f) (Sample B) confirms that the intensity distributions of the In XRF signals coincide well with the peak wavelength distributions of PL in both samples. Examining several intensity profiles (both in the *X* and *Z* directions) in Fig. 3(f), the estimated dimension of the In-rich domains is approximately a few to several tens of microns. So far, co-existence of nanometer- and micrometer-scale potential fluctuations has been suggested in InGaN^23,24^, and this study has visualized the latter fluctuations nondestructively.

Because the XRF measurements detect the amount of In atoms, a larger In XRF signal suggests (1) a wider well width when the In composition is the same and/or (2) a greater In composition when the well width is the same. (Note that because the X-ray can penetrate much deeper than the QW regions, the obtained data are averaged data.) To clarify the dominant cause, APT was applied to Samples A and B. APT can evaluate the interface roughness as well as In segregation of In_*x*_Ga_1-*x*_N with a nanometer resolution^25–28^.

**Figure 4:**
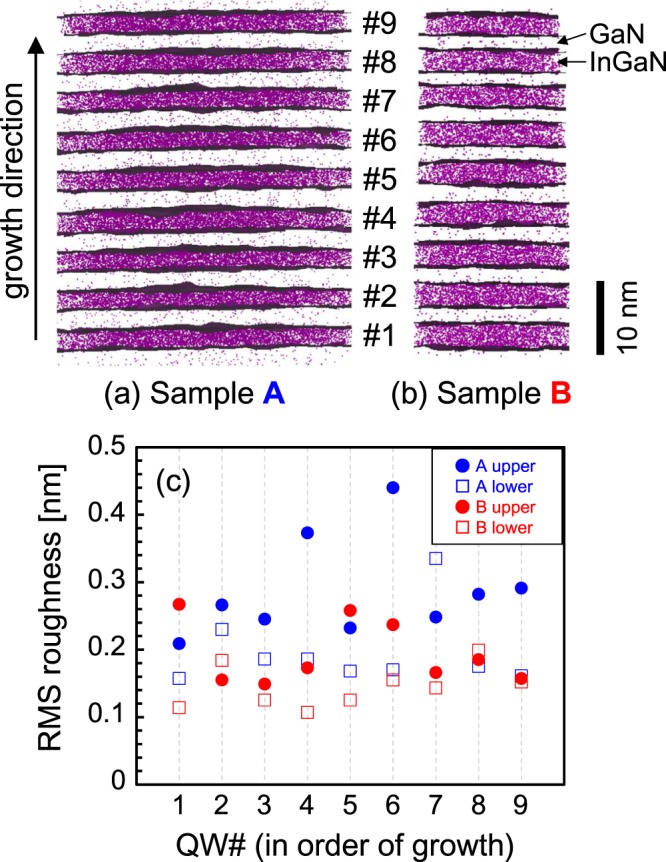
3D APT reconstruction of the In_*x*_Ga_1-*x*_N/GaN QWs for Samples (**a**) A and (**b**) B. Colored dots are In atoms. Gray regions at the In_*x*_Ga_1-*x*_N/GaN interfaces represent 3.0 atom % In isosurfaces in a 1 × 0.5 × 1 nm^3^ voxel size. (**c**) RMS roughnesses of the upper and lower interfaces between In_*x*_Ga_1-*x*_N (well) and GaN (barrier) of each In_*x*_Ga_1-*x*_N layer (#1–9) for both Samples A and B.

APT analysis was performed with a CAMECA LEAP4000X HR and LEAP5000XR atom probe microscope at a sample temperature of 20 K with a laser energy of 10 fJ, a pulse rate of 125 kHz, and a detection rate of 0.5%. Specimen needles for APT were prepared via standard preparation procedures using a dual-beam FIB system (FEI Nova) with a sample lifting up manipulator. The region of interest was defined spanning the QWs as shown in Fig. 4(a,b). This study defined the In_*x*_Ga_1-*x*_N/GaN interface as In_*x*_Ga_1-*x*_N with an In composition of 3% in a 1 × 0.5 × 1 nm^3^ voxel size. In the APT analyses, the direction along the specimen needle usually has a better spatial resolution than that in the perpendicular direction.

To see the In_*x*_Ga_1-*x*_N/GaN interface property in detail, the specimen needle was formed along the [0001] direction. In addition, the voxel size along the growth direction was set to 0.5 nm to precisely evaluate the roughness. The roughness was estimated as the deviation from the ideal flat interface using the root-mean-square (RMS) values. Figure 4(c) shows the interface RMS roughness of both the upper and lower sides of each In_*x*_Ga_1-*x*_N layer (#1–9) for Samples A and B. Here, lower, upper, and the number are defined according to the growth order. The RMS roughness values averaged over all the QWs are 0.287 nm (Sample A: upper), 0.196 nm (Sample A: lower), 0.194 nm (Sample B: upper), and 0.145 nm (Sample B: lower). Sample B has a smaller RMS roughness than Sample A on the nanometer scale, which is likely due to the higher crystal orientation of Sample B, as revealed by the X-ray diffraction line width.

Although APT suggests that the In_*x*_Ga_1-*x*_N/GaN interface properties in Sample B are superior to those in Sample A, XRF indicates that Sample B has a larger In distribution. These findings lead us to the conclusion that the In distribution observed in XRF is not due to fluctuations in the well width but due to fluctuations in the In composition.

From Fig. 3(e,f), the In composition at each measurement point was evaluated as follows. Because the chamber for the sample and detector is purged with He, the In *L* XRF signal emitted from the sample is hardly adsorbed in the atmosphere. For example, under the condition of 295 K, 1 atm, and 1 cm path length, the calculated transmittance of In *L* XRF is 99.98% in He (while 84.75% in air). Therefore, it was assumed that the averaged XRF intensity over all measurement points directly corresponds to the In composition revealed by the macroscopic XRD measurements, which are ~13% for Sample A and ~11% for Sample B. This assumption seems reasonable because the low-energy X-ray (9 keV) can excite the In *L* (~3.3 keV), Al *K* (~1.5 keV), and Ga *L* (~1.2 keV) lines, but re-excitation of the In atoms by the Al *K* or Ga *L* lines does not occur.

Then the XRF signal at each measurement point is directly related to the In composition at that point. The evaluated maximum, minimum, and standard deviation of the In composition in Sample A are 14.3%, 10.5%, and 0.38%, respectively, whereas those in Sample B are 12.8%, 7.8%, and 0.59%, respectively. These results support a larger distribution of the In composition in Sample B than in Sample A.

The exciton localization caused by In fluctuations in the nanometer order is one of the factors that achieves a high luminous efficiency of In_*x*_Ga_1-*x*_N-based LEDs even with dislocation densities of 1 × 10^8^ cm^−2^ (refs^5–9^). On the other hand, as stated above in the discussion of Fig. 3(f), Sample B involves the In distribution on the micrometer order, but has a lower LED output power (Fig. 2(c)). The In distribution on the micrometer order may negatively affect LED characteristics. Therefore, simultaneous control of nanometer-order and micrometer-order fluctuations is required to achieve high device performance. Moreover, the APT measurements suggest better interface abruptness of Sample B than Sample A (Fig. 4). Only making a good interface is not enough to improve LED efficiency.

## Application of SR-XRF to Three-dimensional Structures

Next, the same measurement was applied to a 3D sample. Here, a 3D structured sample means a sample with a nonplanar, three-dimensional surface created by regrowth or post-growth processing. In this study, the sample was fabricated through MOVPE regrowth on stripe SiO_2_ masks. The stripe was along the [1$\bar{1}$00] direction. The mask opening and period were 10 and 20 μm, respectively. The resultant 3D structure consists of (0001), {11$\bar{2}$2}, and {11$\bar{2}$0} facets. Five-period In_*x*_Ga_1-*x*_N/GaN QWs are fabricated on these facets. The designed well and barrier widths are ~3 nm and ~10 nm, respectively, on the (0001) plane. This type of 3D QW structure is suitable for phosphor-free white LEDs because both the inter-facet and intra-facet variations of the In_*x*_Ga_1-*x*_N QW width and In composition realize polychromatic emissions^17–22^.

**Figure 5:**
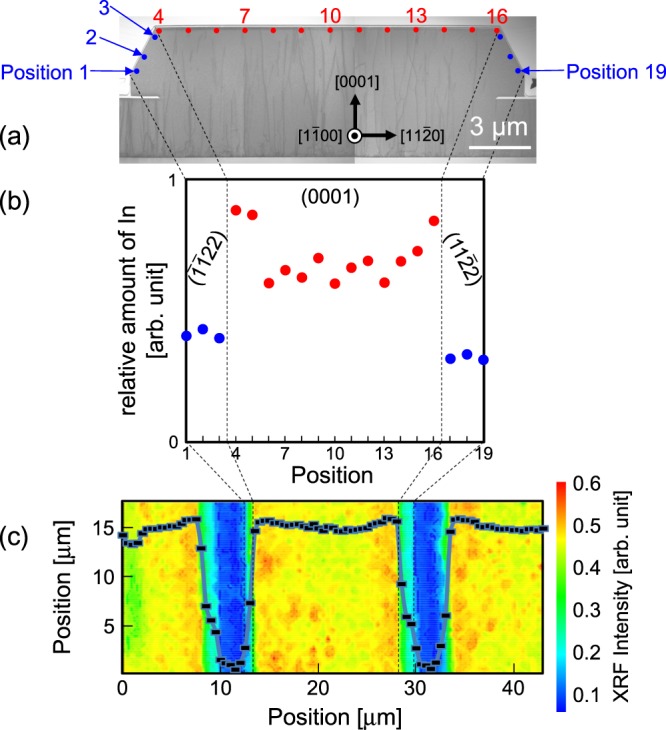
(**a**) Cross sectional TEM image with 3D structure. (**b**) Position dependences of the relative amount of In calculated from the thicknesses and compositions of In_*x*_Ga_1-*x*_N/GaN QWs. Designated numbers (1–19) in (**a**) correspond to the measurement positions in (**b**). (**c**) In *L* XRF intensity map. Scatterplot on the XRF map designates the intensity averaged over all measured points along the [1$\bar{1}$00] direction.

Figure 5(a) shows a cross sectional TEM image, where the QW width and In composition are estimated at positions 1 to 19. Figure 5(b) shows the position-dependent relative amount of In calculated from the equation of (*x* × *t*_InGaN_)/(*t*_InGaN_ + *t*_GaN_). The film thicknesses (*t*_InGaN_, *t*_GaN_) and In composition (*x*) were determined by TEM and EDX analyses. Because the In_*x*_Ga_1-*x*_N thickness and the In composition in the In_*x*_Ga_1-*x*_N/GaN QWs exhibit inter- and intra-facet variations, the relative amount of In changes accordingly.

One concern for XRF measurements is the height variations on the 3D sample surface, which are typically a few μm in this study, because they may lead to issues such as defocusing of the irradiated X-ray beam. Considering the experimental setup, the X-ray beam should be constantly focused to the in-plane size of (*X*, *Z*) = (~0.8 μm, ~0.6 μm) for a distance more than 400 μm along the X-ray passing direction (*Y* direction in Fig. 1(a)). Therefore, defocus of the X-ray beam (and the consequent degradation of the spatial resolution) due to height variations on the sample surface is unlikely. Additionally, the XRF signal is observed nearly parallel to the sample surface by SDD with a diameter of 10 mm, and is unaffected by a few-μm height variation.

For the XRF measurements, the sample was placed on a holder such that the mask stripe is nearly parallel to the XRF detecting direction (i.e., [1$\bar{1}$00] $\parallel$ *X* direction in Fig. 1(a)). With this experimental configuration, the signals from the inclined {11$\bar{2}$2} planes can be detected. The focused X-ray beam was incident along the [0001] direction, and the sample was scanned in 0.5-μm steps along the *X*-*Z* plane. While measuring the inclined (11$\bar{2}$2) plane, the vertical beam size ~0.6 μm is elongated to ~1.1 μm, and the resultant footprint is ~0.8 × ~1.1 μm^2^.

Figure 5(c) shows an In *L* XRF intensity map. The acquisition time to detect the In *L* XRF signal was 5 seconds per point. The scatterplot on the XRF map designates the intensity averaged over all measured points along the mask stripe $\parallel$ [1$\bar{1}$00]. The intensity variation of the In *L* XRF signal coincides with the mask geometry and shows the In distribution. That is, the amount of In on the (0001) plane increases as it approaches the edge of the (0001) plane. This result is consistent with the above TEM-EDX results. Furthermore, the CL intensity maps reported previously^12^ reveal that the emission wavelength around the (0001)-(11$\bar{2}$2) facet boundary is longer than that around the (0001) facet center, which is consistent with the present XRF map. Additionally, in this XRF technique, not only the (0001) plane but also the (11$\bar{2}$2) plane inclined from (0001) can be simultaneously evaluated without changing the experimental setup, which is an advantage over conventional XRD.

## Conclusions

In conclusion, we succeeded in evaluating small In variations of blue LED epitaxial layers using the microbeam XRF technique of the current world’s highest performance synchrotron radiation facility. To use a low-energy X-ray beam to acquire the In *L* XRF signals, the sample chamber was purged with He, mitigating the influence from Ar in air. The In distribution and optical property of In_*x*_Ga_1-*x*_N/GaN QWs are clearly correlated in a μm-scale large area. Although the co-existence of nanometer- and micrometer-scale potential fluctuations has been revealed in InGaN, their roles on device performance have yet to be clarified. This study has demonstrated that microscopic In fluctuations do not necessarily have positive effects on device performance. High performance InGaN-based devices cannot be realized unless not only the nanoscopic In fluctuations, which has thoroughly been studied so far, but also the microscopic In compositional fluctuations are simultaneously and appropriately controlled. The low-energy XRF mapping technique using synchrotron radiation can be a powerful tool for that purpose. Further improvements in the spatial resolution are expected to provide further information on roles of various scale In fluctuations on device performance.
